# National Unified Renal Translational Research Enterprise: Idiopathic Nephrotic Syndrome (NURTuRE-INS) study

**DOI:** 10.1093/ckj/sfae096

**Published:** 2024-03-30

**Authors:** Elizabeth Colby, Samantha Hayward, Melissa Benavente, Fiona Robertson, Agnieszka Bierzynska, Amy Osborne, Kevon Parmesar, Maryam Afzal, Tracey Chapman, Fatima Ullah, Elaine Davies, Michael Nation, Wendy Cook, Tim Johnson, Uwe Andag, Olivier Radresa, Philipp Skroblin, Michaela Bayerlova, Robert Unwin, Nicolas Vuilleumier, Rosamonde E Banks, Fiona Braddon, Ania Koziell, Maarten W Taal, Gavin I Welsh, Moin A Saleem

**Affiliations:** Bristol Renal, Bristol Medical School, University of Bristol, Bristol, UK; Bristol Renal, Bristol Medical School, University of Bristol, Bristol, UK; MRC Integrative Epidemiology Unit, Population Health Sciences, Bristol Medical School, University of Bristol, Bristol, UK; Centre for Kidney Research and Innovation, Academic Unit for Translational Medical Sciences, School of Medicine, University of Nottingham, Nottingham, UK; Centre for Kidney Research and Innovation, Academic Unit for Translational Medical Sciences, School of Medicine, University of Nottingham, Nottingham, UK; Bristol Renal, Bristol Medical School, University of Bristol, Bristol, UK; Bristol Renal, Bristol Medical School, University of Bristol, Bristol, UK; Bristol Renal, Bristol Medical School, University of Bristol, Bristol, UK; Bristol Renal, Bristol Medical School, University of Bristol, Bristol, UK; Bristol Renal, Bristol Medical School, University of Bristol, Bristol, UK; Bristol Renal, Bristol Medical School, University of Bristol, Bristol, UK; Kidney Research UK, Peterborough, UK; Kidney Research UK, Peterborough, UK; Nephrotic Syndrome Trust, Somerset, UK; Experimental Renal Medicine, Medical School, University of Sheffield, Sheffield, UK; Metabolic Disease and Bioinformatics, Evotec International GmbH, Hamburg, Germany; Metabolic Disease and Bioinformatics, Evotec International GmbH, Hamburg, Germany; Metabolic Disease and Bioinformatics, Evotec International GmbH, Hamburg, Germany; Metabolic Disease and Bioinformatics, Evotec International GmbH, Hamburg, Germany; Research and Early Development, Cardiovascular, Renal and Metabolism, BioPharmaceuticals R&D, AstraZeneca, Cambridge, UK; Division of Laboratory Medicine, Diagnostics Department and Department of Medicine Specialties, Geneva University Hospitals and Faculty of Medicine, Geneva, Switzerland; Leeds Institute of Medical Research at St James's, School of Medicine, University of Leeds, Leeds, UK (now Emerita position); UK Renal Registry, Bristol, UK; Department of Experimental Immunobiology, Faculty of Life Sciences and Medicine, King's College London, London, UK; Department of Paediatric Nephrology, Evelina London, Guy's and St Thomas’ NHS Trust, London, UK; Centre for Kidney Research and Innovation, Academic Unit for Translational Medical Sciences, School of Medicine, University of Nottingham, Nottingham, UK; Department of Renal Medicine, Royal Derby Hospital, University Hospitals of Derby and Burton NHS Foundation Trust, Derby, UK; Bristol Renal, Bristol Medical School, University of Bristol, Bristol, UK; Bristol Renal, Bristol Medical School, University of Bristol, Bristol, UK

**Keywords:** biobank, focal segmental glomerulosclerosis, idiopathic nephrotic syndrome, minimal change disease

## Abstract

**Background:**

Idiopathic nephrotic syndrome (INS) is a heterogenous disease and current classification is based on observational responses to therapies or kidney histology. The National Unified Renal Translational Research Enterprise (NURTuRE)-INS cohort aims to facilitate novel ways of stratifying INS patients to improve disease understanding, therapeutics and design of clinical trials.

**Methods:**

NURTuRE-INS is a prospective cohort study of children and adults with INS in a linked biorepository. All recruits had at least one sampling visit collecting serum, plasma, urine and blood for RNA and DNA extraction, frozen within 2 hours of collection. Clinical histology slides and biopsy tissue blocks were also collected.

**Results:**

A total of 739 participants were recruited from 23 centres to NURTuRE-INS, half of whom were diagnosed in childhood [*n* = 365 (49%)]. The majority were white [*n* = 525 (71%)] and the median age at recruitment was 32 years (interquartile range 12–54). Steroid-sensitive nephrotic syndrome (SSNS) was the most common clinical diagnosis [*n* = 518 (70%)]. Of patients diagnosed in childhood who underwent a kidney biopsy, for SSNS (*n* =103), 76 demonstrated minimal change disease (MCD), whereas for steroid-resistant nephrotic syndrome (*n* =80), 21 had MCD. Almost all patients diagnosed in adulthood had a kidney biopsy [*n* = 352 (94%)]; 187 had MCD and 162 had focal segmental glomerulosclerosis.

**Conclusions:**

NURTuRE-INS is a prospective cohort study with high-quality biosamples and longitudinal data that will assist research into the mechanistic stratification of INS. Samples and data will be available through a Strategic Access and Oversight Committee.

## Graphical Abstract

**Graphical Abstract:**
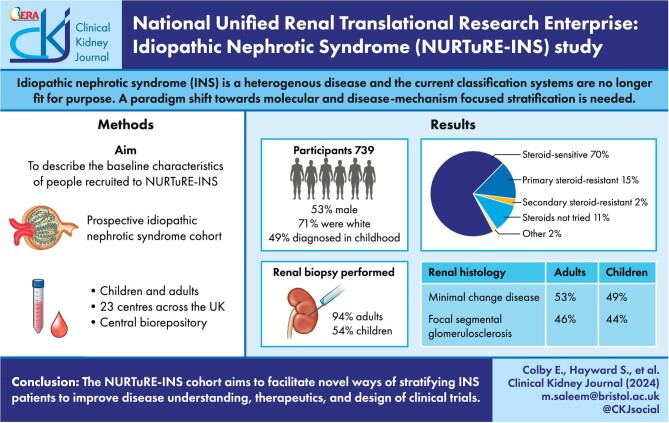

KEY LEARNING POINTS **What was known:**

- Idiopathic nephrotic syndrome (INS) is a heterogenous disease and the current INS classification systems are no longer fit for purpose. A paradigm shift towards molecular and disease-mechanism focused stratification is needed.

**This study adds:**

- This article provides a description of the NURTuRE-INS cohort, which consists of high-quality biosamples and detailed clinical data for 739 INS patients from across the UK.
- When comparing adult and paediatric recruits, the proportion of patients in the different clinical and histological subgroups is highly comparable.
- Paediatric recruits had a higher estimated glomerular filtration rate at baseline than adult recruits.

**Potential impact:**

- The NURTuRE-INS cohort will facilitate research into the mechanistic underpinnings of INS.
- Novel ways to subgroup INS patients would allow current treatments to be used more effectively as well as help development of new therapies.

## INTRODUCTION

Nephrotic syndrome (NS) causes a breakdown of the glomerular filtration barrier, leading to a loss of essential circulating proteins into the urine. NS is a heterogenous disease with different clinical classification criteria used in children and adults. Paediatric patients are grouped by their response to steroid treatment, whereas adult patients are classified by renal histology results [1]. For all patients, treatment regimens centre on broad immunosuppression, which can be accompanied by significant side effects. It is evident that a change in the stratification of NS is long overdue. Novel subgroups defined by disease mechanism and consistent across paediatric and adult populations are needed. This would allow current treatments to be used in a targeted manner, reducing unnecessary exposure to potentially harmful medications and reducing the costs associated with ineffective treatment. Additionally the development of new therapies and stratified clinical trials would be facilitated.

Our current understanding of the underlying disease pathways in NS, segregates patients into three broad groups:

• Monogenic NS: At least 33% of cases of childhood-onset steroid-resistant NS (SRNS) are caused by pathogenic variants in single genes. To date, causative variants in >60 genes have been identified in SRNS [2].
• Circulating factor disease (CFD) NS: A separate subset of patients who exhibit disease recurrence after transplantation are believed to have CFD. This theory is supported by both observational and laboratory work; serum or plasma from CFD patients can induce specific effects on cell lines and in animal models [3–8].
• Currently undefined: There are patients with both steroid-sensitive NS (SSNS) and SRNS that do not clearly fit into one of the above two categories. Screening of a UK cohort with SRNS revealed 26.2% had monogenic NS and 13.8% had probable CFD, leaving more than half of patients as undefined [9]. Whether these undefined patients are a single mechanistic subgroup, possibly with SSNS and SRNS lying at different ends of a spectrum, or whether they split into smaller distinct subgroups is also unknown.

To improve clinical care and for patients to benefit, these NS groups must be better defined. Through the collection of detailed clinical information and biological samples, the NURTuRE-INS cohort aims to facilitate a paradigm shift to a more molecular- and disease-mechanism-focused stratification of NS that will facilitate research to develop and test novel therapies.

## MATERIALS AND METHODS

### Study design

NURTuRE-INS is a prospective multicentre observational study created through collaboration between academic investigators, industry partners and Kidney Research UK. At present, NURTuRE consists of two projects, NURTuRE INS and NURTuRE chronic kidney disease (NURTuRE-CKD) [10]. Through these projects, NURTuRE has provided a template for kidney research in the UK, including a national network of research nurses for patient recruitment to both cohorts, a centralized biorepository for optimal sample storage and an independent Strategic Oversight and Access Committee (SOAC). The SOAC oversees the wider research community's access to the patient samples and the sharing of any analyses performed on the samples.

### Eligibility and enrolment

Patient recruitment was carried out from September 2017 to February 2023. Participants were recruited from 15 adult and 8 paediatric nephrology clinics at secondary care sites in England, Wales and Scotland (Supplementary Table 1). Participants were approached if they had a diagnosis of congenital NS and childhood or adult-onset NS with primary SRNS, late onset of steroid resistance, SSNS, INS as part of a syndrome (e.g. nail patella syndrome and Denys–Drash syndrome), focal segmental glomerulosclerosis (FSGS) or minimal change disease (MCD). Patients were excluded if they had a secondary cause of NS, such as vasculitis, diabetes, obesity or hypertension. Patients (or, if the patient was <18 years of age, their guardian) provided written informed consent to participate in both the NURTuRE-INS study and the UK Kidney Association National Registry of Rare Kidney Diseases (RaDaR) study [11].

### Data management and ethical approval

The UK Renal Registry (UKRR) via the RaDaR database houses the clinical data for study recruits [12] and includes ongoing automated routine laboratory data uploads. The NURTuRE-INS study (09/H0106/80) and RaDaR study (14/SW/1088) were approved by the South-West - Central Bristol Ethics Committee.

### Baseline visits and sample and data collection

Baseline information was collected by research nurses from participants’ medical records, study interviews and questionnaires. Clinical diagnoses were obtained from the medical records and histological diagnoses from renal biopsy reports. In addition, all patients had sociodemographic details, past medical history, family history and medication data collected, as well as height, weight and blood pressure readings. Quality-of-life data were collected using validated age-appropriate questionnaires. Quality of life [European Quality of Life 5-Level 5-Dimension questionnaire (EQ-5D-5L)], symptoms (IPOS-Renal), anxiety and depression [Hospital Anxiety and Depression Scale (HADS)] and cognition [6-item Cognitive Impairment Test (6-CIT)] scores were completed for adult patients [13–16]. Quality of life (EQ-5D-5L for ≥16 years and EQ-5D-7 for 7–15 years) and health-related quality of life [Child Health Utility 9D (CHU9D), for 7–17-year-olds] questionnaires were completed for paediatric patients [17]. Retrospective and prospective clinical information, including routine blood and urine results, hospital admissions and details of renal replacement therapies and death, will also be available through the UKRR and NHS Digital.

Blood and urine samples for the biobank were collected from all participants. If patients had undergone a clinical biopsy, processed histology slides and a surplus biopsy tissue block were collected. If patients had undergone plasma exchange treatment for a relapse of NS, the waste plasma exchange fluid was collected.

### Follow-up visits, samples and data collection

Disease-dependent follow-up visits were conducted if a patient experienced a relapse (2 visits—1 in relapse, 1 in remission) or underwent transplantation (5 visits—1 week before, 1 week after and then at 6 monthly intervals). Additional routine follow-up visits were carried out at least 6 months after the baseline visit, for one-third of the study cohort. The same data and samples that were collected at the baseline study visit were repeated at the follow-up visits. Prospective clinical information will be collected automatically through the UKRR and NHS Digital, allowing long-term follow-up of participants.

### Sample processing and storage

Venous blood for plasma (ethylenediaminetetraacetic acid and lithium heparin anticoagulants), serum (clot activator tubes) and whole blood for DNA and RNA extraction, together with mid-stream urine (complete and centrifuged) samples were collected and stored securely in multiple aliquots at −80°C. Detailed standard operating procedures were written and site visits conducted to ensure standardization across all sites (see Supplementary method 1). For patients who underwent a renal biopsy as part of their routine care, stored haematoxylin and eosin, periodic acid–Schiff and trichrome stained slides and formalin fixed paraffin embedded (FFPE) tissue cores were collected. Sections were cut from surplus tissue in the FFPE block for additional staining, RNA extraction and proteomic analysis.

### Laboratory analysis

Routine blood and urine testing was performed at local hospital laboratories. Centralized biomarker analysis was performed at University Hospitals Geneva, Geneva, Switzerland (see Supplementary Table 2 for the list of 27 biomarkers). Exome sequencing was performed by AstraZeneca on an HLI-NovaSeq 6000 (Illumina, San Diego, CA, USA). DNA methylation analysis using the Illumina EPIC array was performed by the University of Bristol. RNA sequencing was performed by Evotec (see Supplementary method 1). A summary of the data generated is provided in Supplementary Table 3.

### Study management scheme

A joint steering committee (JSC), including academics, Kidney Research UK and industry representatives, met quarterly to provide oversight for the study and to review progress. Top-level oversight of the biorepository patient samples was provided by the SOAC, which was independent of the JSC.

### Data analysis

Descriptive statistics have been used to summarize the baseline NURTuRE data. Estimated glomerular filtration rate trajectory (eGFR slope) and time-averaged urine albumin:creatinine ratio (UACR) prior to study recruitment (baseline) were calculated for patients. eGFR was calculated using the Bedside Schwartz equation for childhood measurements and the 2021 Chronic Kidney Disease Epidemiology Collaboration equation for adult datapoints (without ethnicity adjustment). Unadjusted linear regression was used to calculate the eGFR slope using the linear model lm function from the stats package in R (see Supplementary Method 2). UACR data were available for 251 patients; urine protein:creatinine ratio data were available for a further 320 patients and used to estimate UACR [18]. Time-averaged UACR was calculated for each patient (see Supplementary Method 2 for details). For all analyses, R version 4.1.1 (R Foundation for Statistical Computing, Vienna, Austria) was used.

## RESULTS

### Demographics

In total, 739 participants were recruited to NURTuRE-INS, half of whom were diagnosed with NS in childhood [*n* = 365 (49%)]. The time between diagnosis and recruitment into the study varied (Supplementary Fig. 2); 18 patients (2%) were recruited within 2 weeks of diagnosis and 139 (19%) were recruited within the first year of diagnosis. By 1 February 2024, the median follow-up time since the first study visit was 5 years, however, follow-up of participants continues automatically through UKRR and NHS Digital. A summary of the baseline clinical and demographic characteristics of the study cohort are presented in Table 1 (patients diagnosed in childhood) and Table 2 (patients diagnosed in adulthood). The majority of the cohort are of white ethnicity [*n* = 525 (71%)] and there was a roughly equal split between genders [males, *n* = 390 (53%)]. The median age at recruitment was 32 years [interquartile range (IQR) 12–54] and the median age at diagnosis was 19 years (IQR 4–42). A greater proportion of patients recruited from England were from areas of high deprivation [*n* = 169 (26%) in index of multiple deprivation quintile (IMDQ) 1]. However, the inverse was seen in the small number of patients recruited in Scotland, with more patients recruited from the least deprived area [IMDQ 5; Scotland, *n* = 9 (36%)]. For Wales, near equal numbers of patients were recruited from areas of high deprivation [IMDQ 1; Wales, *n* = 15 (22%)] to those of least deprivation [IMDQ 5; Wales, *n* = 16 (24%)]. Information on additional sociodemographic characteristics of the patients diagnosed in adulthood are available in Supplementary Table 4.

**Table 1:** Baseline characteristics—patients diagnosed with INS in childhood.

Characteristics	Overall (*N* = 365)	SSNS [*n* = 252 (69%)]	PSRNS [*n* = 72 (20%)]	SSRNS [*n* = 15 (4%)]	INS, steroids not tried [*n* = 22 (6%)]	Denys–Drash syndrome [*n* = 3 (1%)]	INS, unknown response to steroids [*n* = 1 (<1%)]
Sex, *n* (%)							
Male	201 (55)	152 (60)	31 (43)	7 (47)	8 (36)	2 (67)	1 (100)
Female	164 (45)	100 (40)	41 (57)	8 (53)	14 (64)	1 (33)	0
Ethnicity, *n* (%)							
White	223 (61)	146 (58)	52 (72)	9 (60)	12 (55)	3 (100)	1 (100)
Asian (Pakistani, Indian or Bangladeshi background)	80 (22)	59 (23)	11 (15)	5 (33)	5 (23)	0	0
Black African, Black Caribbean or other Black background	29 (8)	24 (10)	2 (3)	1 (7)	2 (9)	0	0
Mixed ethnicity	22 (6)	14 (6)	6 (8)	0	2 (9)	0	0
Other ethnicity	4 (1)	2 (<1)	1 (1)	0	1 (5)	0	0
Asian (Chinese or other Asian background)	2 (<1)	2 (<1)	0	0	0	0	0
Patient declined to answer	5 (1)	5 (2)	0	0	0	0	0
IMDQ (England), *n* (%)							
1	109 (32)	82 (34)	13 (20)	2 (14)	12 (60)	0	0
2	65 (19)	44 (18)	14 (22)	5 (36)	0	1 (33)	1 (100)
3	47 (14)	29 (12)	10 (15)	2 (14)	5 (25)	1 (33)	0
4	63 (18)	45 (19)	10 (15)	4 (29)	3 (15)	1 (33)	0
5	57 (17)	38 (16)	18 (28)	1 (7)	0	0	0
Age at diagnosis (years), median (IQR)	3 (2–7)	4 (2–7)	5.0 (2–9)	2.0 (2–4)	0 (0–3)	1.0 (1–2)	13
Age at recruitment (years, median (IQR)	12 (7–19)	12 (7–21)	12 (7–16)	12 (9–16)	12 (6–19)	7 (4–12)	64
Dialysis at recruitment, *n* (%)	8 (2)	1 (<1)	4 (6)	2 (13)	0	1 (33)	0
Kidney transplant at recruitment, *n* (%)	30 (8)	2 (<1)	16 (22)	1 (7)	8 (36)	2 (67)	1 (100%)
Transplant recurrence prior to recruitment, *n* (%)	9 (30)	0	7 (44)	1 (100)	1 (13)	0	0
Renal biopsy performed, *n* (%)	197 (54)	103 (41)	67 (93)	13 (87)	10 (45)	3 (100)	1 (100)
Histological diagnosis, *n* (%)							
MCD	97 (49)	76 (74)	15 (22)	6 (46)	0	0	0
FSGS	87 (44)	25 (24)	48 (72)	7 (54)	6 (60)	0	1 (100)
Other	13 (7)	2 (2)	4 (6)	0	4 (40)	3 (100)	0
Medications at recruitment, *n* (%)							
Corticosteroids	142 (39)	100 (40)	29 (40)	6 (40)	6 (27)	0	1 (100)
Calcineurin inhibitors	117 (32)	67 (26)	38 (53)	5 (33)	7 (32)	0	0
Mycophenolate mofetil	76 (21)	49 (19)	21 (29)	3 (20)	3 (14)	0	0
Cyclophosphamide	3 (<1)	3 (1)	0	0	0	0	0
Golimumab or adalimumab	1 (<1)	1 (<1)	0	0	0	0	0
Rituximab	9 (3)	8 (3)	1 (1)	0	0	0	0
Pazopanib	0	0	0	0	0	0	0
Warfarin, direct oral anticoagulant or heparin	10 (3)	3 (1)	4 (6)	0	1 (5)	1 (33)	1 (100)
Antiplatelet	12 (3.3)	5 (2)	4 (6)	0	2 (9)	0	1 (100)
Statin	25 (6.8)	12 (5)	8 (11)	1 (7)	3 (14)	0	1 (100)
Renin–angiotensin system inhibitor	55 (15)	28 (11)	17 (24)	2 (13)	8 (36)	0	0

**Table 2:** Baseline characteristics—patients diagnosed with INS in adulthood.

Characteristics	Overall (*N* = 374)	SSNS [*n* = 266 (71%)]	PSRNS [*n* = 36 (10%)]	SSRNS [*n* = 4 (1%)]	INS, steroids not tried [*n* = 60 (16%)]	INS, unknown response to steroids [*n* =8 (2%)]
Sex, *n* (%)						
Male	189 (51)	131 (49)	18 (50)	2 (50)	32 (53)	6 (75)
Female	185 (49)	135 (51)	18 (50)	2 (50)	28 (47)	2 (25)
Ethnicity						
White	302 (81)	215 (81)	29 (81)	3 (75)	50 (83)	5 (62)
Asian (Pakistani, Indian or Bangladeshi background)	39 (10)	28 (11)	4 (11)	1 (25)	4 (7	2 (25)
Black African, Black Caribbean or other Black background	20 (5)	11 (4)	3 (8)	0	5 (8)	1 (12)
Mixed ethnicity	2 (<1)	2 (<1)	0	0	1 (1.7)	0
Other ethnicity	5 (1)	4 (2)	0	0	0	0
Asian (Chinese or other Asian background)	5 (1)	5 (2)	0	0	0	0
Patient declined to answer	1 (<1)	1 (<1)	0	0	0	0
IMDQ (England),, *n* (%)						
1	60 (20)	43 (19)	11 (33)	0	5 (12)	1 (17)
2	54 (18)	37 (17)	6 (18)	1 (25)	8 (20)	2 (33)
3	68 (22)	51 (23)	6 (18)	1 (25)	10 (24)	0
4	62 (20)	43 (19)	5 (15)	1 (25)	11 (27)	2 (33)
5	62 (20)	48 (22)	5 (15)	1 (25)	7 (17)	1 (17)
Age at diagnosis (years), median (IQR)	42 (30–58)	46 (31–60)	38 (28–45)	38 (34–44)	37 (26–51)	32 (29–40)
Age at recruitment (years), median (IQR)	52 (39–65)	54 (40–66)	45 (35–54)	53 (42–63)	52 (39–62)	49 (37–60)
Dialysis at recruitment, *n* (%)	11 (3)	5 (2)	1 (3)	0	5 (8)	0
Kidney transplant at recruitment, *n* (%)	28 (8)	8 (3)	5 (14)	0	11 (18)	4 (50)
Transplant recurrence prior to recruitment, *n* (%)	1 (<1)	1 (<1)	0	0	0	0
Renal biopsy performed, *n* (%)	352 (94)	255 (96)	33 (92)	4 (100)	52 (87)	8 (100)
Histological diagnosis, *n* (%)						
MCD	187 (53)	168 (66)	11 (33)	2 (50)	5 (10)	1 (12)
FSGS	162 (46)	84 (33)	22 (67)	2 (50)	47 (90)	7 (88)
Other	3 (<1)	3 (1)	0	0	0	0
Medications at recruitment, *n* (%)						
Corticosteroids	126 (34)	108 (41)	13 (36)	0	2 (3)	3 (38)
Calcineurin inhibitors	103 (28)	72 (27)	13 (22)	13 (36)	2 (50)	3 (38)
Mycophenolate mofetil	25 (7)	12 (5)	4 (11)	1 (25)	6 (10)	2 (25)
Cyclophosphamide	2 (<1)	1 (<1)	1 (3)	0	0	0
Golimumab or adalimumab	1 (<1)	1 (<1)	0	0	0	0
Rituximab	1 (<1)	1 (<1)	0	0	0	0
Pazopanib	1 (<1)	0	1 (3)	0	0	0
Warfarin, direct oral anticoagulant or heparin	33 (9)	23 (9)	5 (14)	0	2 (3)	2 (25)
Antiplatelet	49 (13)	34 (13)	6 (17)	0	7 (12)	2 (25)
Statin	133 (36)	91 (34)	17 (47)	1 (25)	22 (37)	2 (25)
Renin–angiotensin system inhibitor	195 (52)	133 (50)	22 (61)	2 (50)	34 (57)	4 (50)

### Disease severity and treatment

At the time of recruitment, 58 patients (8%) had received a kidney transplant and 19 patients (3%) were on dialysis. Of the 662 (90%) patients who had not started renal replacement therapy, the median eGFR at recruitment was 95 ml/min/1.73 m^2^ (data available for 604 patients), the median kidney function trajectory (eGFR slope) prior to the first study visit was −1.04 mL/min/1.73 m^2^/year (data available for 501 patients) and the median time-averaged UACR was 87.54 mg/mmol (data available for 535 patients; Table 3).

**Table 3:** Baseline kidney function.

	All patients (*N* = 739)		Patients not on RRT only (*n* = 662)		
Group	Median eGFR slope prior to recruitment or RRT start (ml/min/1.73 m^2^/year)	Median time-averaged UACR prior to recruitment or RRT start (ml/min/1.73 m^2^/year)	Median eGFR at recruitment (ml/min/1.73 m^2^)	Median eGFR slope prior to recruitment (ml/min/1.73 m^2^/year)	Median time-averaged UACR prior to recruitment (mg/mmol ^)^
All (*N* = 739)	−1.31 (*n* = 542)	100.85 (*n* = 570)	94.49 (*n* = 604)	−1.04 (*n* = 501)	87.54 (*n* = 535)
Adult onset (*n* = 374)	−2.3 (*n* = 310)	96.08 (*n* = 291)	77.44 (*n* = 329)	−0.77 (*n* = 287)	84.94 (*n* = 274)
Childhood onset (*n* = 365)	−1.77 (*n* = 232)	107.7 (*n* = 279)	117.53 (*n* = 275)	−1.22 (*n* = 214)	97.12 (*n* = 261)
Steroid resistant (*n* = 127)	−4.98 (*n* = 92)	286.44 (*n* = 96)	94.72 (*n* = 88)	−3.14 (*n* = 77)	261.5 (*n* = 79)
Steroid sensitive (*n* = 518)	−0.36 (*n* = 386)	68.76 (*n* = 419)	95.89 (*n* = 458)	−0.23 (*n* = 376)	66.73 (*n* = 413)

At the baseline study visit, roughly one-third of patients had been prescribed a calcineurin inhibitor [*n* = 220 (30%)] and/or corticosteroids [*n* = 268 (36%)]. The next most commonly prescribed immunosuppressive medication was mycophenolate mofetil [*n* = 101 (14%)]. Prescriptions for all other immunosuppression were rare, with <1.5% of the study cohort on any of these medications.

### NS subgroups—clinical and histological classifications

**Figure 1:**
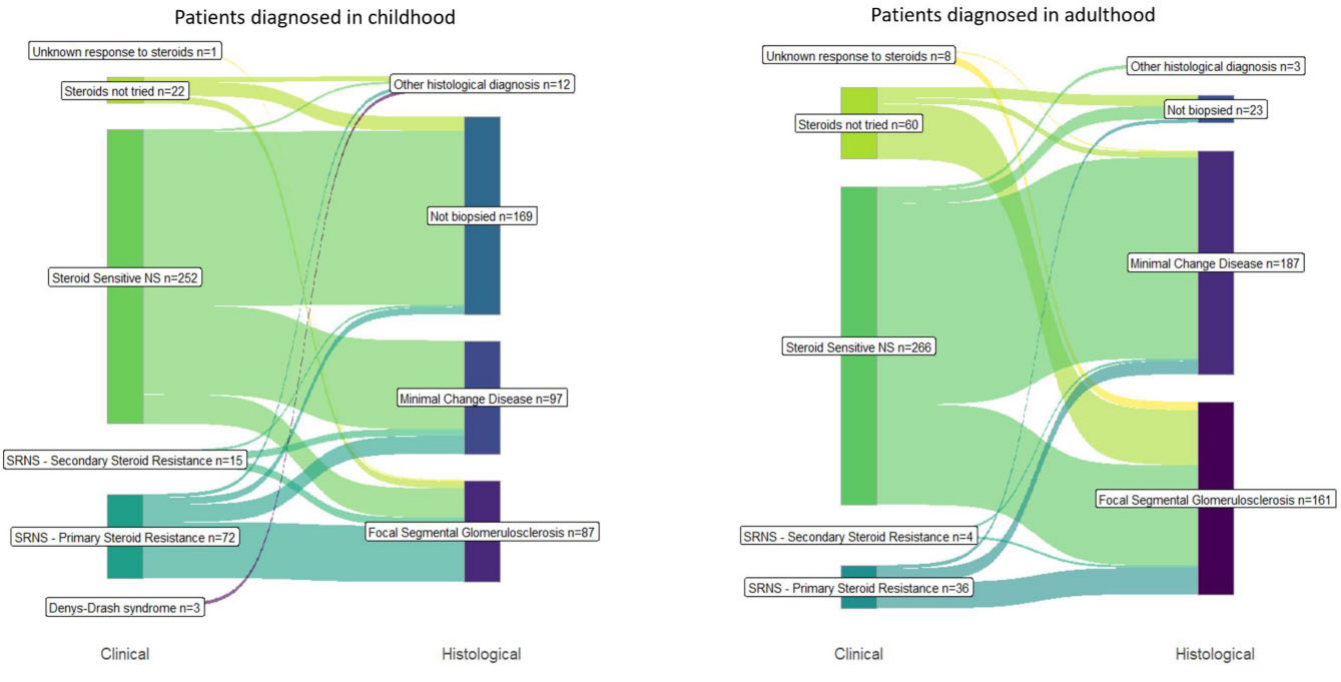
Crossover between clinical and histological INS classification.

The clinical diagnostic groups that are used in paediatrics do not cleanly overlap with the histological diagnoses that are commonplace in adult nephrology, as illustrated in Fig. 1. Although steroid sensitivity more commonly corresponded to MCD on renal biopsy for both children and adults, there was still a significant number of people who were steroid sensitive with FSGS on biopsy or were steroid resistant with MCD on biopsy.

Overall, SSNS was the most common clinical diagnostic subgroup for patients diagnosed in childhood and adulthood [*n* = 518 (70%)]. Information on steroid response was unavailable for nine patients (1%), as they had either been diagnosed in a different country or >20 years ago. Of the 252 SSNS patients diagnosed in childhood, 38 (15%) were classed as having frequently relapsing SSNS, 50 (20%) were steroid dependent and 11 (4%) had partial steroid resistance (Table 4). A total of 103 of the paediatric SSNS patients underwent a kidney biopsy; 76 (74%) demonstrated MCD and 25 (24%) had FSGS. An additional 15 paediatric patients were steroid sensitive at the time of diagnosis but subsequently developed steroid resistance (secondary SRNS); 7 (54%) of these patients had FSGS on biopsy and 6 (46%) had MCD.

**Table 4:** Adult versus childhood comparison of SSNS categories.

Subcategories of steroid-sensitive diagnosis	Diagnosed in childhood (*n* = 252)	Diagnosed in adulthood (*n* = 266)
Frequently relapsing, *n* (%)	38 (15)	31 (12)
Steroid dependent, *n* (%)	50 (20)	24 (9)
Partial steroid resistance, *n* (%)	11 (4)	24 (9)
Steroid sensitive—no further information, *n* (%)	153 (61)	187 (70)

Kidney Disease: Improving Global Outcomes subcategory definitions: steroid dependent—two consecutive relapses during therapy with prednisone or prednisolone (either at full dose or during tapering) or within 15 days of prednisone or prednisolone discontinuation; frequently relapsing—two or more relapses per 6 months within 6 months of disease onset or four or more relapses per 12 months in any subsequent 12-month period.

Of the patients who were diagnosed with SSNS in adulthood (*n* = 266), 31 (12%) had frequent relapses, 24 (9%) were steroid dependent and 24 (9%) had partial steroid resistance. An additional four adult patients had secondary SRNS. Almost all adult patients underwent a kidney biopsy (*n* = 352); of those with SSNS, 168 (66%) had MCD and 84 (33%) had FSGS, while for patients with primary SRNS, FSGS was more common [*n* = 22 (67%)] than MCD [*n* = 11 (33%)]. Of the small number of patients who had secondary SRNS, an equal number demonstrated MCD [*n* = 2 (50%)] and FSGS [*n* = 2 (50%)]. A subgroup of patients (*n* = 60) did not receive steroid treatment at diagnosis and most of these patients had FSGS on biopsy (*n* = 47 of 52 who underwent a biopsy (90%)].

### NURTuRE-INS compared with other large national and international NS cohorts

A summary of NURTuRE-INS compared with other NS cohorts is presented in Table 5. Even though half of the NURTuRE-INS cohort were diagnosed with NS in childhood, the majority of the cohort were recruited when they were ≥18 years of age [*n* = 469 (63%)]. Despite this, when compared with the other cohorts in Table 5, NURTuRE-INS still has the most balanced recruitment of adults and children with NS. The International Study of NS (International NephroS) has the same inclusion and exclusion criteria as NURTuRE-INS, allowing for the data from these cohorts to be cleanly combined and for meaningful comparisons to be made between the cohorts, particularly in reference to ethnicity. Also of note, four of the cohorts [NURTuRE-INS, NephroS, the NS study Network (NEPTUNE) and Insight into NS (INSIGHT)] have collected and stored blood, urine and DNA samples from patients. This opens the door for collaboration between cohorts and independent replication and validation of key findings.

**Table 5:** Characteristics of NURTuRE-INS compared with other large national or international NS cohorts (*N* > 500)

Cohort	NURTuRE-INS	International study of NS (International NephroS)	NS Study Network (NEPTUNE)	Insight into NS (INSIGHT)	European Rare Kidney Disease Reference Network (ERKNet)
Country	UK	India, Sri Lanka and South Africa	USA	Canada	Europe
NS patients, *n*					
Total	739	892	729	631	1 062 869 (82%)
By age at recruitment:					
Adults (≥18 years)	469 (63%)	70 (8%)	355 (49%)	Not recruited	
Children (<18 years)	270 (37%)	822 (92%)	374 (51%)^a^	631 (100%)^b^	220 (21%)^c^
Inclusion criteria	Congenital NS, SSNS, SRNS (primary resistance), SRNS (secondary resistance), INS as part of a syndrome, FSGS or MCD	Congenital NS, SSNS, SRNS (primary resistance), SRNS (secondary resistance), INS as part of a syndrome, FSGS or MCD	FSGS, MCD, membranous nephropathy or non-biopsied and treatment naïve paediatric patients with NS	Incident diagnosis of NS and age 6 months–18 years	Congenital NS, congenital NS—Finnish type, SSNS, SRNS (secondary resistance), genetic SRNS, SRNS (sensitive to second-line immunosuppression), SRNS (multidrug resistant) or NS not otherwise specified. Other rare non-NS disease groups are also recruited
Exclusion criteria	Secondary NS	Secondary NS	Prior solid organ transplant, evidence of other renal disease, known systemic disease with life expectancy <6 months	Secondary NS, congenital NS, syndromic disease with multiple organ involvement	
Data and sample collection	Sociodemographic and clinical data	Sociodemographic and clinical data	Sociodemographic and clinical data	Sociodemographic and clinical data.	Sociodemographic and clinical data
	Blood, DNA, RNA, urine and renal histology samples as well as waste plasma exchange fluid	Blood, DNA, RNA and urine samples	Blood, DNA, urine and renal histology samples	Blood, DNA, urine and toenail clipping samples	
Recruitment started	2017	2017	2010	2011	2018
Recruitment completed	2023	2022	Ongoing—estimated 2024	Ongoing	Ongoing
Follow-up	At disease relapse or transplantation.	At disease relapse or transplantation	Minimum of 30 months	5 years	Ongoing—minimum of annual data collection.
	At least 6 months after recruitment.				
	Automated prospective follow-up of routine clinical information and testing—ongoing				
Access to data and samples	Applications for sample access can be made to the SOAC via Kidney Research UK	Not explicitly stated	Applications for data and sample access can be made via the NEPTUNE website (https://www.neptune-study.org/ancillary-studies)	Not explicitly stated	Applications for data access can be made via the registry website (https://www.erknet.org/patients-registry/data-access-requests)

Patient numbers came from ^a^personal communication, ^b^Prediction of Short- and Long-Term Outcomes in Childhood Nephrotic Syndrome (sickkids.ca) and ^c^https://www.erknet.org/.

## DISCUSSION

For the majority of patients, the disease mechanism driving NS is unknown. The identification of causative pathogenic genetic variants in a small subset of patients with NS has improved our understanding of the disease. However, further work to characterize the remaining NS patients is needed. In this article, we have outlined the baseline characteristics and the samples collected from the NURTuRE-INS cohort. The comprehensive multi-omics data generated from the patient biosamples, alongside the robust and detailed clinical data in this cohort, will serve as a key resource to explore the elusive molecular mechanisms of NS. Additionally, the exome sequencing data will allow patients with monogenic NS to be identified, as well as searching for novel disease-modifying variants.

In the NURTuRE-INS cohort, the clinical diagnostic categories that are used in paediatric nephrology showed minimal overlap with the histological groupings used in adult nephrology. Interestingly, however, when we apply the same subclassification used in paediatrics to the adult cohort (Tables 1, 2 and 4), the proportion of patients across those categories is very similar in both, perhaps signifying mechanistic overlap. This illustrates the need for a novel strategy to define these diseases, reinforcing the importance of NURTuRE-INS and other large NS studies that are trying to establish novel molecular subgroups and biomarkers. A stratification system that is disease-mechanism focused and unified across paediatric and adult patients is the ultimate goal. As NURTuRE-INS has recruited a fairly even number of adults and children, this cohort may be best placed to offer unique insights into the pathogenesis of NS at different ages. For example, previously it had been expected that monogenic NS would present exclusively in childhood. However, there is increasing evidence of people with underlying pathogenic genetic variants developing NS in adulthood. In a cohort of patients with adult-onset SRNS or biopsy-proven FSGS with no family history of NS, 12% of patients had a pathogenic NS variant [19]. In addition, when a cohort of adults with CKD of unknown cause but with other affected family members were screened, 5% had pathogenic NS variants [20]. It is plausible that distinct disease mechanisms are at play at different stages of the life course. However, on balance, it is more likely that similar mechanisms are driving disease, particularly for younger adults and children.

The NURTuRE-INS cohort has a good balance of genders but limited ethnic diversity; the majority of the cohort is White, 16% of recruits are from South Asian backgrounds and 7% from Black backgrounds. People from South Asian and Black backgrounds are more likely to develop NS and less likely to respond to first-line treatment [21–23]. Some of the disparity observed for Black patients may be due to the higher frequency of G1 and G2 *APOL1* risk alleles in patients of recent sub-Saharan or West African ancestry [24]; children with a high-risk *APOL1* genotype have a faster decline in their kidney function and are less likely to achieve complete remission of proteinuria [25, 26]. Unfortunately, the NURTuRE-INS cohort probably contains an insufficient number of patients from these ethnic backgrounds to draw any meaningful biological insights into the adverse outcomes experienced by these patient groups. To address this limitation, our team created the International NephroS cohort, which recruits patients from India, Sri Lanka and South Africa. By combining data from NURTuRE-INS and International NephroS we hope to be able to explore whether the differences in outcomes between ethnicities are due to genetic, psychosocial or other molecular drivers.

Through the creation of NURTuRE and its first two cohorts (INS and CKD), we hope to provide a resource for multi-omics renal research in the UK and accelerate the mechanistic stratification of kidney disease. Creation of the NURTuRE biorepository for long-term storage of samples and the SOAC for sample access oversight ensures that we have high-quality patient samples available for potential future collaborations. Data sharing and collaboration in rare diseases such as INS are crucial; NURTuRE-INS adds an additional NS cohort that can be used for independent replication or validation of any promising research findings. Additionally, because of the ability of the UKRR to upload laboratory data both retrospectively (as far back as 1995) and prospectively, we offer the possibility to link disease-specific data such as proteinuria with long-term clinical outcomes such as kidney failure; this will be important in the future design of interventional clinical trials.

A potential limitation of NURTuRE-INS is that patients were recruited at any stage of their disease rather than at diagnosis. As a result, patients have different durations of follow-up and have had a variety of exposure to treatments. Also, as the study is observational, the histological diagnoses are only available for patients who have undergone a kidney biopsy as part of their routine clinical care. For paediatric patients, it is likely that those who have had a biopsy represent a group with more complicated and treatment-resistant disease. Therefore, their biopsy results should not be interpreted as representative of the whole cohort of paediatric patients. However, the key strengths of NURTuRE include the robust high standard of sample collection and storage and the depth of clinical data collected for each patient. Furthermore, through automated electronic linkage with the UKRR and NHS Digital, both retrospective and prospective clinical data are available for patients.

NURTuRE-INS is a prospective multicentre observation study with detailed clinical information and high-quality biosamples. The study aims to recategorize NS into distinct mechanistic disease groups, leading to current treatments being used more effectively, as well as the development of new and better-targeted therapies and stratified clinical trials. Applications for access to the stored biosamples can be made to the SOAC through Kidney Research UK.

## Supplementary Material

sfae096_Supplemental_Files

## Data Availability

Anonymised participant-level data will be made available to external investigators upon successful application to the SOAC.
