# Supplementary material for: National Unified Renal Translational Research Enterprise: Idiopathic Nephrotic Syndrome (NURTuRE-INS) study
Source: Clin Kidney J. 2024 Mar 30;17(8):sfae096. doi: 10.1093/ckj/sfae096 (PMC11317841; doi:10.1093/ckj/sfae096)
Supplement: sfae096_Supplemental_Files [file sfae096_supplemental_files.zip › 118 suppl figures.pptx]

## Slide 1
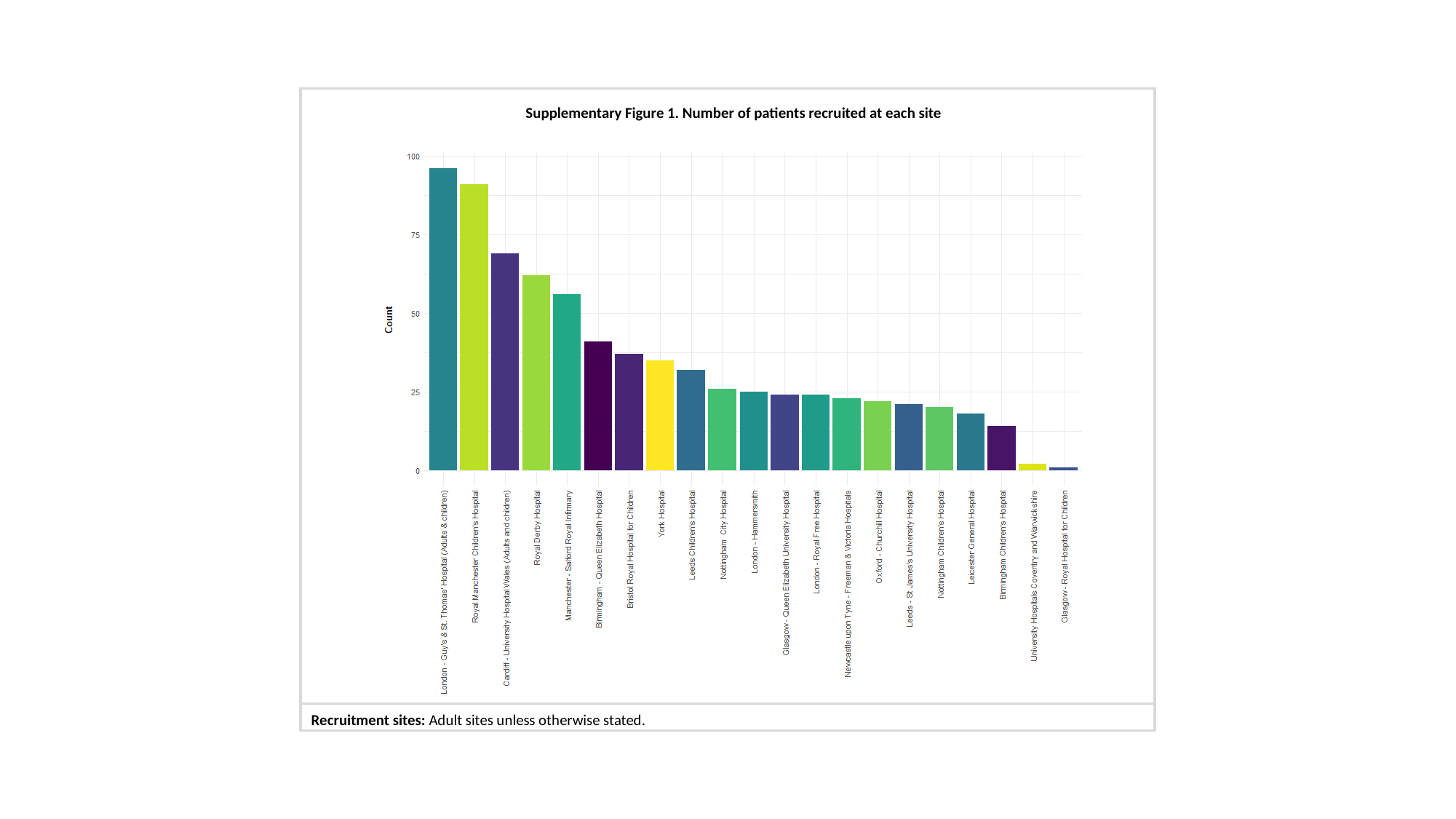

Supplementary Figure 1. Number of patients recruited at each site
Recruitment sites: Adult sites unless otherwise stated.

## Slide 2
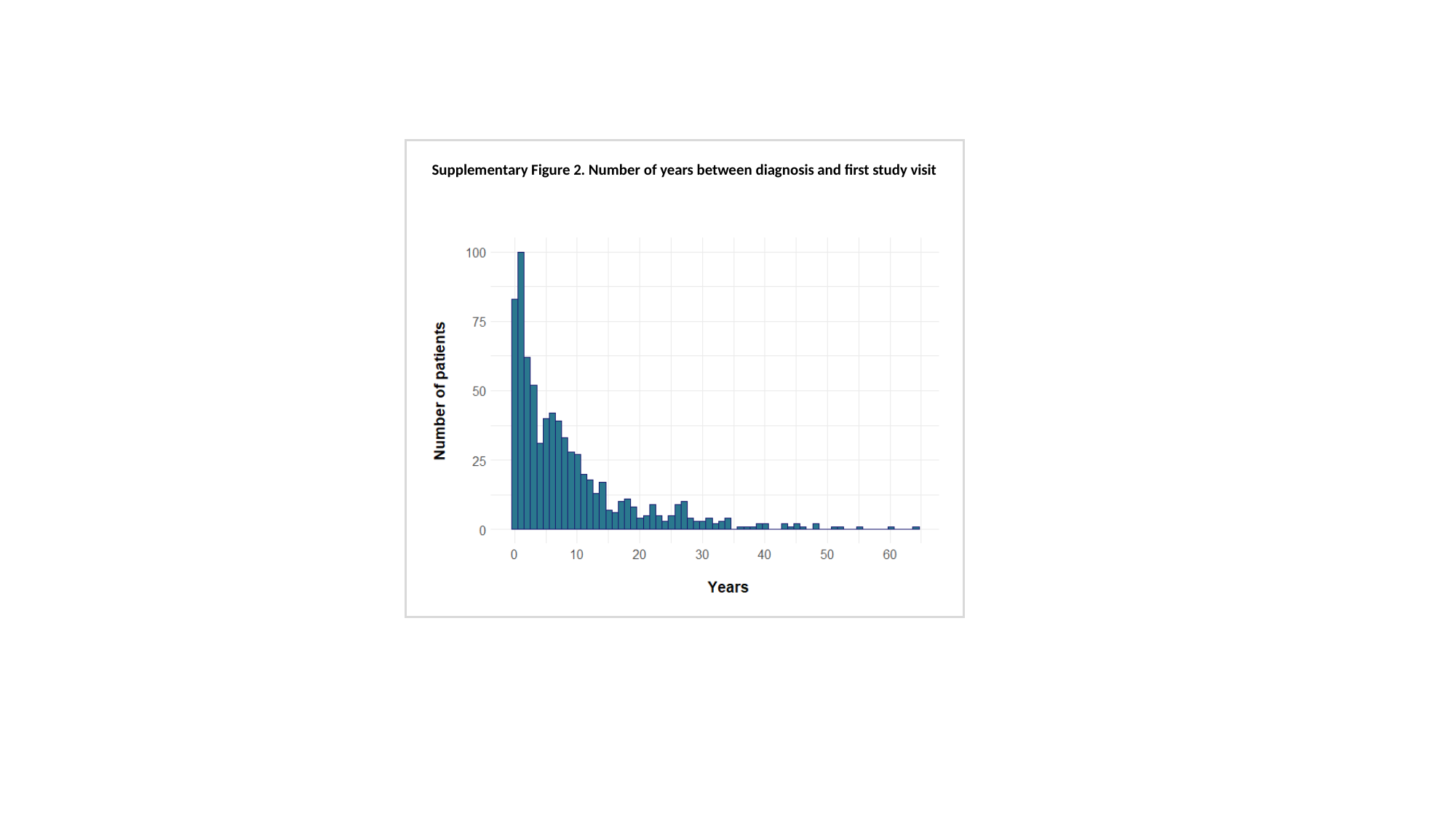

Supplementary Figure 2. Number of years between diagnosis and first study visit
