# Supplementary material for: National Unified Renal Translational Research Enterprise: Idiopathic Nephrotic Syndrome (NURTuRE-INS) study
Source: Clin Kidney J. 2024 Mar 30;17(8):sfae096. doi: 10.1093/ckj/sfae096 (PMC11317841; doi:10.1093/ckj/sfae096)
Supplement: sfae096_Supplemental_Files [file sfae096_supplemental_files.zip › INS baseline paper Supplementary CKJ revised.docx]

| **Supplementary Table 1. UK Recruitment centres and number of participants** | | | | |
| --- | --- | --- | --- | --- |
| **Centre** | **City** | **Principal Investigator** | **Research Nurses/ Co-ordinators** | **Enrolment** |
| Queen Elizabeth Hospital | Birmingham | P. Hewins | M. Dutton  N. Walmsley  L. Fifer  K. Kuningas | 41 |
| Birmingham Children’s hospital | Birmingham | L. Kerecuk | M. Kokocinska  S. Parks | 14 |
| Bristol Royal Hospital for Children | Bristol | M. Saleem | R. Helyer  S. Dymond  S. Turner | 37 |
| University Hospital Wales | Cardiff | S. Griffin | A. Hole  R. Rencricca  M. Coleman  G. Nicol  Y. Webley | 60 |
| Children’s Hospital for Wales | Cardiff | S. Hegde | J. Muller  Z. Morrison | 9 |
| University Hospitals Coventry and Warwickshire | Coventry | W. Ayub | S. Hewins  G. Evans | 2 |
| Royal Derby Hospital | Derby | M. Taal | K. White  S. Hussain | 62 |
| Queen Elizabeth University Hospital | Glasgow | P. Mark | R. McDougall  N. Parker | 24 |
| Royal Hospital for Children | Glasgow | B. Reynolds | K. McLaughlin  N. MacDonald | 1 |
| St James’s University Hospital | Leeds | A. Lewington | K. Tobin  S. Dorey | 21 |
| Leeds Children’s Hospital | Leeds | K. Tyerman | J. Clark  C. Spencer  L. Wright | 32 |
| Leicester General Hospital | Leicester | J. Barratt | S. Budwal | 18 |
| Hammersmith Hospital | London | M. Griffith | R. Almasarwah  M. Ijeomah-Orji | 25 |
| Royal Free Hospital | London | R. Pepper | P. Kaur Babooa  R. Davies  P. Gardner | 24 |
| Guy’s and St. Thomas’ Hospital | London | A. Koziell | K. Pastou  C. Briggs | 52 |
| Evelina London Children’s Hospital | London | A. Koziell | K. Pastou  C. Briggs | 44 |
| Salford Royal Infirmary | Manchester | P. Kalra | C. Summersgill  M. Beswick  S. Whittaker | 56 |
| Royal Manchester Children’s Hospital | Manchester | R. Lennon | A. Branson  C. Prakash  A. Cocker-Swanick | 91 |
| Freeman and Victoria Hospitals | Newcastle Upon Tyne | J. Sayer | C. Mather  L. Langhorne  L. Hutchinson  R. Bradbury | 23 |
| Nottingham City Hospital | Nottingham | M. Hall | S. Brand  S. Haslam  N. Straw  J. Agbonmwandolor | 26 |
| Nottingham Children’s Hospital | Nottingham | M. Christian | N. Khan  L. Lawless | 20 |
| Churchill Hospital | Oxford | K. Bull | M. Weetman  K. Parsons  R. Rabara  M. Gavrila | 22 |
| York Hospital | York | C. Jones | J. Anderson  S. Sutton | 35 |

| **Supplementary Table 2. List of biomarkers measured in clinical fluids** | |
| --- | --- |
| **Protein biomarker** | **Sample** |
| Albumin | Complete urine |
| Calbindin | Complete urine |
| CD40 | Lithium-heparin plasma |
| CD40 ligand | Lithium-heparin plasma |
| Clusterin | Complete urine |
| Collagen type 1 alpha 1 chain | Complete urine |
| Creatinine | Serum and complete urine |
| C reactive protein | Serum |
| Cystatin C | Serum |
| Fibroblast growth factor-23 | Lithium-heparin plasma |
| Galectin-3 | Lithium-heparin plasma |
| Growth/differentiation factor-15 | Serum |
| High-sensitivity troponin T | Serum |
| Interleukin-17A | Lithium-heparin plasma |
| Interleukin-1 beta | Lithium-heparin plasma |
| Interleukin-6 | Lithium-heparin plasma |
| Kidney Injury Molecule-1 | Lithium-heparin plasma and complete urine |
| Monocyte chemoattractant protein-1 | Lithium-heparin plasma |
| Matrix metallopeptidase 9 | Complete urine |
| Neutrophil gelatinase-associated lipocalin | Lithium-heparin plasma |
| N-terminal pro B-type natriuretic peptide | Serum |
| Osteoactivin | Complete urine |
| Soluble urokinase plasminogen activator receptor | EDTA plasma |
| Tissue inhibitor of metalloproteinase-1 | Complete urine |
| Tumor necrosis factor alpha | Lithium-heparin plasma |
| Tumor necrosis factor receptor 1 | Lithium-heparin plasma |
| Vascular endothelial growth factor | Complete urine |

| **Supplementary Table 3. Samples collected and data generated** | | |
| --- | --- | --- |
| **Sample type** | **Tissue type** | **Data generated** |
| Routine clinical samples | Blood | Full Blood Count, Urea and Electrolytes, Estimated Glomerular Filtration Rate (eGFR), Serum Creatinine, Magnesium, Calcium, Phosphate, Serum Albumin, Bicarbonate, Uric acid, C-Reactive Protein (CRP), Serum Parathyroid hormone, Lipid Profile (including triglycerides), Glucose, HbA1c (if diabetic), Ferritin, Folic Acid, Vitamin B12 (If anaemic). |
|  | Urine | Urine Albumin Creatinine Ratio (UACR), Urine Albumin, Urine Creatinine. |
| Stored research samples | Plasma, serum and urine | 27 biomarkers (listed in Supplementary Table 2). |
|  | DNA | Exome sequencing - HLI-NovaSeq6000.  SNP array - Illumina Global Screening array v2.0 with additional multi-disease content and 2k custom sequences.  Epigenetic DNA methylation – Illumina EPIC array. |
|  | RNA | RNA sequencing (blood) - Sequencing libraries were prepared using the Tecan Universal Plus mRNA kit with custom globin and mitochondrial-genome encoded gene depletion. Pooled libraries were sequenced on an Illumina NovaSeq 6000 system. |
|  | FFPE tissue | Glomerular proteomic profiles  Brightfield images  Multiplex Images  Single cell transcriptomics  Spatial transcriptomics |

| **Supplementary Table 4. Additional socio-demographic characteristics - patients diagnosed in adulthood** | | | | | | |
| --- | --- | --- | --- | --- | --- | --- |
| **Characteristic** | **Overall, N = 374** | **Steroid-Sensitive NS, N = 266** | **Primary Steroid-Resistant NS, N = 36** | **Secondary Steroid-Resistant NS, N = 4** | **INS - Steroids not tried, N = 60** | **INS - unknown response to steroids , N = 8** |
| **Smoking** |  |  |  |  |  |  |
| Never smoked | 210 (56%) | 155 (58%) | 20 (56%) | 2 (50%) | 28 (47%) | 5 (62%) |
| Ex-Smoker | 122 (33%) | 85 (32%) | 12 (33%) | 2 (50%) | 21 (35%) | 2 (25%) |
| Smoker | 32 (9%) | 18 (7%) | 3 (8%) | 0 (0%) | 10 (17%) | 1 (12%) |
| Missing data | 10 (3%) | 8 (3%) | 1 (3%) | 0 (0%) | 1 (2%) | 0 (0%) |
| **Alcohol use** | 170 (45%) | 125 (47%) | 13 (36%) | 4 (100%) | 25 (42%) | 3 (38%) |
| Missing data | 11 (3%) | 9 (3%) | 1 (3%) | 0 | 1 (2%) | 0 |
| **Diet** |  |  |  |  |  |  |
| No restrictions | 315 (84%) | 230 (86%) | 28 (78%) | 4 (100%) | 48 (80%) | 5 (62%) |
| Vegetarian | 17 (5%) | 12 (5%) | 0 (0%) | 0 (0%) | 3 (5%) | 2 (25%) |
| Pescatarian | 15 (4%) | 9 (3%) | 2 (6%) | 0 (0%) | 4 (7%) | 0 (0%) |
| Vegan | 4 (1%) | 2 (<1%) | 1 (3%) | 0 (0%) | 1 (2%) | 0 (0%) |
| Low Protein | 2 (<1%) | 1 (<1%) | 0 (0%) | 0 (0%) | 1 (2%) | 0 (0%) |
| Other | 13 (4%) | 6 (2%) | 4 (11%) | 0 (0%) | 2 (3%) | 1 (12%) |
| Missing data | 8 (2%) | 6 (2%) | 1 (3%) | 0 (0%) | 1 (2%) | 0 (0%) |
| **Marital status** |  |  |  |  |  |  |
| Married or Civil Partner | 225 (60%) | 157 (59%) | 21 (58%) | 4 (100%) | 37 (62%) | 7 (88%) |
| Single | 85 (23%) | 65 (24%) | 6 (17%) | 0 (0%) | 14 (23%) | 1 (12%) |
| Divorced or dissolved Civil Partnership | 31 (8%) | 21 (8%) | 7 (19%) | 0 (0%) | 3 (5%) | 0 (0%) |
| Widowed or surviving Civil Partner | 14 (4%) | 9 (3%) | 1 (3%) | 0 (0%) | 4 (7%) | 0 (0%) |
| Separated | 8 (2%) | 7 (3%) | 1 (3%) | 0 (0%) | 0 (0%) | 0 (0%) |
| Missing data | 9 (2%) | 7 (3%) | 0 (0%) | 0 (0%) | 2 (3%) | 0 (0%) |
| **Highest educational attainment** |  |  |  |  |  |  |
| None | 42 (11%) | 31 (12%) | 2 (6%) | 0 (0%) | 8 (13%) | 1 (12%) |
| General Certificate of Secondary Education | 87 (23%) | 64 (24%) | 8 (22%) | 2 (50%) | 13 (22%) | 0 (0%) |
| National Vocational Qualifications | 77 (21%) | 54 (20%) | 11 (31%) | 0 (0%) | 11 (18%) | 1 (11%) |
| A Level | 26 (7.0%) | 20 (8%) | 2 (6%) | 0 (0%) | 3 (5%) | 1 (11%) |
| Undergraduate degree | 75 (20%) | 51 (19%) | 8 (22%) | 1 (25%) | 13 (22%) | 2 (25%) |
| Postgraduate degree | 54 (14%) | 35 (13%) | 4 (11%) | 1 (25%) | 11 (18%) | 3 (38%) |
| Other | 4 (1%) | 4 (2%) | 0 (0%) | 0 (0%) | 0 (0%) | 0 (0%) |
| Missing data | 9 (2%) | 7 (3%) | 1 (2.8%) | 0 (0%) | 1 (2%) | 0 (0%) |
| **Employment** |  |  |  |  |  |  |
| Student | 3 (<1%) | 3 (1%) | 0 (0%) | 0 (0%) | 0 (0%) | 0 (0%) |
| Unemployed | 11 (3%) | 4 (2%) | 2 (6%) | 0 (0%) | 4 (7%) | 1 (12%) |
| Unable to work due to health reasons | 20 (5%) | 11 (4%) | 5 (14%) | 0 (0%) | 3 (5%) | 1 (12%) |
| Unable to work due to family or caring responsibilities | 11 (3%) | 9 (3%) | 0 (0%) | 0 (0%) | 2 (3%) | 0 (0%) |
| Not in work for another reason | 6 (2%) | 5 (2%) | 1 (3%) | 0 (0%) | 0 (0%) | 0 (0%) |
| Working part time | 55 (15%) | 42 (16%) | 5 (14%) | 0 (0%) | 8 (13%) | 0 (0%) |
| Working full time | 159 (43%) | 105 (39%) | 16 (44%) | 3 (75%) | 31 (52%) | 4 (50%) |
| Retired from paid work | 101 (27%) | 80 (30%) | 7 (19%) | 1 (25%) | 11 (18%) | 2 (25%) |
| Missing data | 8 (2%) | 7 (3%) | 0 (0%) | 0 (0%) | 1 (2%) | 0 (0%) |
| **First language** |  |  |  |  |  |  |
| English | 329 (88%) | 232 (87%) | 33 (92%) | 3 (75%) | 55 (92%) | 6 (75%) |
| Punjabi | 6 (2%) | 5 (2%) | 0 (0%) | 0 (0%) | 1 (2%) | 0 (0%) |
| Gujarati | 3 (<1%) | 3 (1%) | 0 (0%) | 0 (0%) | 0 (0%) | 0 (0%) |
| Portuguese | 3 (<1%) | 2 (<1%) | 1 (3%) | 0 (0%) | 0 (0%) | 0 (0%) |
| Urdu | 3 (<1%) | 2 (<1%) | 0 (0%) | 0 (0%) | 1 (2%) | 0 (0%) |
| Chinese | 2 (<1%) | 2 (<1%) | 0 (0%) | 0 (0%) | 0 (0%) | 0 (0%) |
| Polish | 2 (<1%) | 2 (<1%) | 0 (0%) | 0 (0%) | 0 (0%) | 0 (0%) |
| Tamil | 2 (<1%) | 1 (<1%) | 0 (0%) | 0 (0%) | 0 (0%) | 1 (12%) |
| Other | 10 (3%) | 4 (2%) | 2 (6%) | 1 (25%) | 2 (3%) | 1 (12%) |
| Missing data | 14 (4%) | 13 (5%) | 0 (0%) | 0 (0%) | 1 (2%) | 0 (0%) |
| **Literacy** |  |  |  |  |  |  |
| Good fluency | 344 (92%) | 242 (91%) | 34 (94%) | 4 (100%) | 56 (93%) | 8 (100%) |
| Moderate fluency | 12 (3%) | 12 (5%) | 0 (0%) | 0 (0%) | 0 (0%) | 0 (0%) |
| Weak fluency | 5 (1%) | 2 (<1%) | 1 (3%) | 0 (0%) | 2 (3%) | 0 (0%) |
| Unable to speak or read | 3 (<1%) | 2 (<1%) | 0 (0%) | 0 (0%) | 1 (2%) | 0 (0%) |
| Missing data | 10 (3%) | 8 (3%) | 1 (3%) | 0 (0%) | 1 (2%) | 0 (0%) |

**Supplementary methods**

^1^ Patient samples were barcoded, with a unique identifier per patient/per visit and linked back to study ID number. All urine samples and plasma blood tubes were collected and immediately placed on ice. BD Bioscience Vacutainer tubes were supplied to all sites. For serum and Li-hep plasma, a mix of collection tubes containing separator gel and no gel were used. For adult patients up to 70mls of blood was collected at each sampling timepoint. For children the amount of blood collected per visit varied by size of child. For patients <15kg, up to 6mls of blood was collected. For patients between 15-30kg, up to 12.5mls of blood and for patients >30kg, up to 24mls of blood. Serum blood tubes were left to clot for 20 minutes at room temperature before being placed on ice. Samples were centrifuged at 4 ^o^C, 1200 rcf for 10 minutes, sample was pooled if multiple collection tubes were used and then aliquoted and stored at -80 ^o^C. Samples were stored at -80 ^o^C within 2 hours of collection and any deviations from this recorded. Blood samples for DNA and RNA extraction were left at room temperature for 2-24 hours, transferred to -20 ^o^C for 24-96 hours and then stored at -80 ^o^C. Periodic shipments of samples on dry ice were made from collection sites to the biorepository at National Institute of Health Research (NIHR), National Biosample Centre, Milton Keynes, UK. Waste plasma exchange bags were stored and transported chilled, before being aliquoted and stored at -80 ^o^C. All -80 ^o^C freezers had electronic temperature monitoring. Renal histology samples were sent to the Human Biomaterials Resource Centre (HBRC) the University of Birmingham. The stained slides (or slides cut from the blocks and subsequently stained) were digitally scanned. For the RNA sequencing, libraries were prepared using the Tecan Universal Plus mRNA kit with custom globin and mitochondrial-genome encoded gene depletion. Pooled libraries were sequenced on Illumina NovaSeq 6000 system.

^2^For patients who had not started RRT, the eGFR from the day of recruitment or closest measurement taken within 30 days of recruitment (n=604) was used as the baseline value. Patients with at least two eGFR measurements (n=542) had eGFR slopes calculated; the slopes were censored at recruitment date (n=662), or start of RRT date for those who commenced RRT prior to the baseline study visit (n=77). For the time average uACR, this was calculated for each patient by dividing the area under the curve along the x-axis of time from first measurement to baseline date or renal replacement therapy date, whichever was soonest, and y-axis of uACR, by the total time. The area under the curve was computed using the ‘auc’ function in the flux package in R.

**Supplementary figures**

**Supplementary Figure 1. Number of patients recruited at each site**

**Recruitment sites:** Adult sites unless otherwise stated.


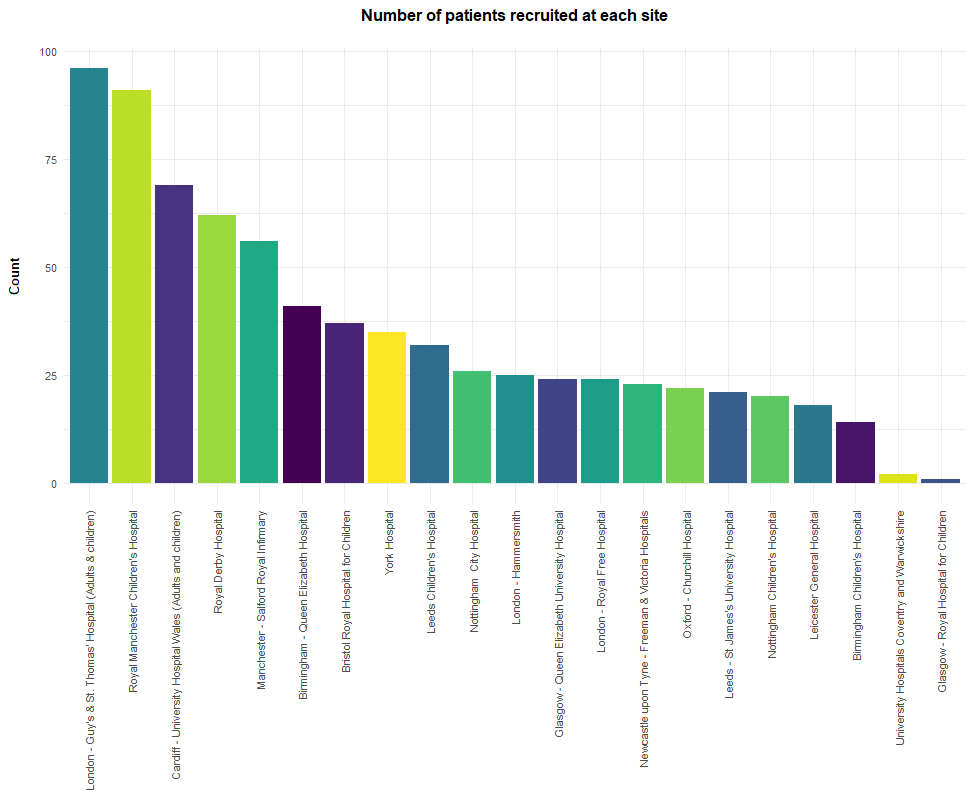

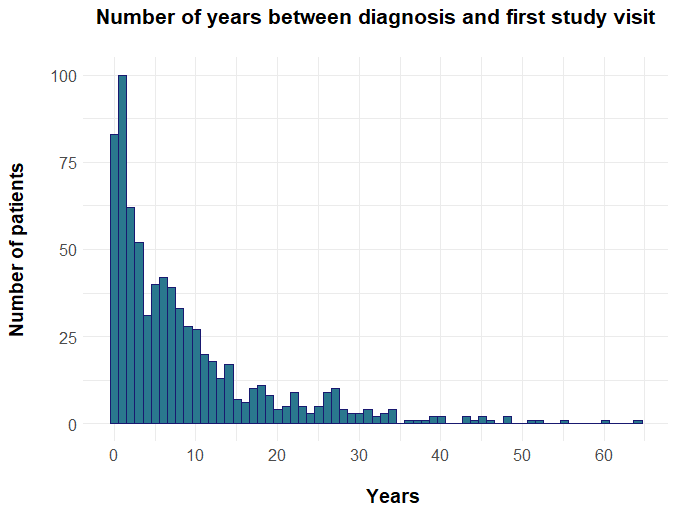


**Supplementary Figure 2. Number of years between diagnosis and first study visit**
